# An Overview of the Cardiorespiratory Hypothesis and Its Potential Contribution to the Care of Neurodegenerative Disease in Africa

**DOI:** 10.3390/medicina55090601

**Published:** 2019-09-17

**Authors:** Nounagnon Frutueux Agbangla, Sarah A. Fraser, Cédric T. Albinet

**Affiliations:** 1 Unité de Recherche Pluridisciplinaire Sport Santé Société (URePSSS-EA 7369), Univ. Artois, Univ. Lille, Univ. Littoral Côte d’Opale, F-59000 Lille, France; 2 Interdisciplinary School of Health Sciences, Faculty of Health Sciences, University of Ottawa, Ottawa, ON K1S 5S9, Canada; sarah.fraser@uottawa.ca; 3 Laboratoire Sciences de la Cognition, Technologie, Ergonomie (SCoTE-EA7420), Université de Toulouse, INU Champollion, 81012 Albi, France; cedric.albinet@univ-jfc.fr

**Keywords:** cardiorespiratory hypothesis, Africa, neurodegenerative disease

## Abstract

One hypothesis that could explain the beneficial effects of physical exercise on cognitive function is the cardiorespiratory hypothesis. This hypothesis proposes that improved cognitive functioning may be in part a result of the physiological processes that occur after physical exercise such as: Increased cerebral perfusion and regional cerebral blood flow. These processes ensure increased oxygenation and glucose transportation to the brain, which together can improve cognitive function. The objective of this narrative review is to examine the contribution of this hypothesis in the care of African older adults with neurodegenerative conditions (i.e., dementia (Alzheimer’s disease)) or with mild cognitive impairments. Although studies in developed countries have examined people of African descent (i.e., with African Americans), only the limited findings presented in this review reflect how these conditions are also important for the African continent. This review revealed that no studies have examined the effects of cardiorespiratory fitness on neurodegenerative disease in Africa. African nations, like many other developing countries, have an aging population that is growing and will face an increased risk of neurodegenerative declines. It is therefore imperative that new research projects be developed to explore the role of the cardiorespiratory fitness in neurodegenerative disease prevention in African nations.

## 1. Introduction

Africa is considered a continent with a young population. It is estimated that the percentage of people under 25 years will be 59.43% by 2020 [1]. Despite this fact, the number of older adults (>60 years) increases every year according to global health (the World Health Organization; WHO) and the local health promotion/prevention initiatives [2]. For instance, in 2005, the number of people in Africa over 60 years was 53 million and it is expected that this number will reach 200 million in 2050 [3]. While health promotion/prevention initiatives have reduced infectious conditions in low- and middle-income countries, non-communicable diseases (i.e., cardiovascular disease) are on the rise [4]. As a consequence of this, Africa will face important health changes and challenges associated with individuals who are aging in the upcoming decades. 

Aging has been defined as the gradual decline of biological functions caused by progressive dysfunction of different cellular systems responsible for repairing and maintaining the homeostasis [5]. This gradual decline, along with other risk factors, can result in the emergence of Mild Cognitive Impairment (MCI; memory decline without pathology) and neurodegenerative pathologies such as dementia and Alzheimer’s disease (AD). The issue of age-related changes and its deleterious effects on cognitive health in Africa is not well documented. The importance of understanding the existing literature and preparing for a population shift in Africa towards older adults cannot be underestimated.

The few studies addressing these issues have focused on determining the risk factors and the prevalence rate of MCI and age-related cognitive pathologies (dementia and AD) [6,7,8,9,10,11,12,13,14]. In the case of MCI, data on prevalence are limited, as only a few studies have been conducted in smaller African countries. The results of these studies have indicated that the prevalence of MCI is 10.4% in rural areas of Benin [6] and 10.8% in Senegal [14]. Risk factors for the development of MCI in these populations are: Poor social network, heart disease, stroke, epilepsy, head trauma, and family history [14]. In addition, other factors such as depression and lack of apolipoprotein ε2 have been highlighted [6]. Regarding dementia, data from several African countries are available. For example, the prevalence of dementia is 8.87% in Senegal [13], 2.6% in rural areas [6] and 3.7% in urban Benin [12], 10.1% in Nigeria [9], 8.1% in the Central African Republic, and 6.7% in the Republic of Congo [7]. Risk factors are gender (women are generally more affected), hypertension, low body mass index (<18.5 Kg/m^2^), depressive symptoms, and a low level of education [8]. However, a recent study in South Africa found that dementia was not associated with gender nor with education when the age of the participants was controlled [15]. Apart from these main risk factors, other diseases, which are prominent in Africa such as hypertension, diabetes, hypercholesterolemia, and stroke have been shown to contribute to an increase in the prevalence of dementia [15,16].

In summary, the prevalence of dementia in Africa is growing and is moving towards rates seen in developed countries. This prevalence, which seems low in comparison to developed countries, will increase in the coming years because the population is living longer. Indeed, it is predicted that by 2050, 71% of people with dementia will live in low- and middle-income countries [17]. Finally, very little work has been carried out regarding AD in Sub-Saharan Africa. Only risk factors such as age and gender [9], and the presence of apolipoprotein ε4 (APOE ε4) homozygote [10], are well documented. In addition to these studies conducted in Sub-Saharan Africa, other studies have been carried out in North Africa demonstrating that the prevalence increases as a function of age in Egypt going from 2.26% in people over 50 years to 18.48% among people over 80 and over [18]. The presence of APOE ε4 has also been identified as a risk factor in the North African population [19]. The synthesis of these studies confirms that neurodegenerative pathologies linked to aging are present in low rates on the continent. Several factors can explain this low rate such as differential survival rates, unreported cases, and poor access to medical care [20]. In order to manage these age-related diseases, it seems important to develop appropriate strategies. These strategies should integrate several domains including: Medicine, psychology, and sports science. 

These findings of cognitive impairment, dementia, and AD are important because physical exercise might be the answer to improving or maintaining cognitive health in this population. Physical exercise remains one of the most robust strategies that contribute to improving the functioning of the brain, helps to prevent cognitive impairment, and reduces the incidence of AD [21]. Findings of a recent meta-analysis demonstrated that low energy expenditure values between 0–2000 Kcal/week or 0–45 Met-h/week were associated with a higher risk of dementia and AD. In contrast, an increase of 500 Kcal/week or 10 Met-h/week of this energy expenditure through leisure activities decreases the risk of dementia and AD by 13% and 10%, respectively [22]. Among the many biological mechanisms underlying this prophylactic effect, an increase in growth factors such as Insulin Growth Factor-1 (IGF-1) and Vascular Endothelial Growth Factor (VEGF) are the most reported [21]. Other mechanisms such as growth of vessels in the hippocampus, the cortex, and the cerebellum, the increased production of nitric oxide (NO) and increased cerebral blood flow and oxygen delivery can help to explain this effect [21]. All these mechanisms are components of an explanatory hypothesis called the cardiorespiratory hypothesis [23]. The cardiorespiratory hypothesis helps explain how these mechanisms may relate to (a) a reduction in cognitive decline and (b) improved cognitive function in an aging population. 

The aims of this narrative review are first to present the cardiorespiratory hypothesis. The second aim of this review is to discuss studies examining the effects of cardiorespiratory fitness on MCI, dementia, and Alzheimer’s disease. Finally, we aimed to explore how the cardiorespiratory hypothesis may apply to neurodegenerative diseases in Africa.

## 2. What Is the Cardiorespiratory Hypothesis?

This hypothesis, which was proposed for the first time by Dustman and collaborators [23], suggests that physical exercise, particularly aerobic exercise, which increases cardiorespiratory fitness, increases cerebral perfusion and regional cerebral blood flow [24]. This induces increased oxygen and glucose transportation to the brain, which results in improved cognitive functioning [25,26]. Several biological mechanisms can underlie this hypothesis. One mechanism is angiogenesis, which is defined as a primary mechanism of formation of new capillaries from existing blood vessels [27]. Beyond the developmental period, the formation of new capillaries in the brain may occur under the influence of the stimulation of physical exercise [28]. Indeed, regular physical exercise can induce the proliferation of a protein called vascular endothelial growth factor (VEGF), which is the main precursor of angiogenesis [24]. However, the peptide hormone insulin-like growth factor one (IGF-1) secreted by the liver also plays an important role in the mechanism of angiogenesis. This peptide hormone promotes the growth of the cerebral blood vessels and influences the regulation of VEGF [29]. Evidence of this angiogenesis mechanism has been supported by the work carried out in animals. In rodents, regular voluntary physical exercise increases angiogenesis in the motor cortex [30], in the dentate gyrus [31], and finally in the cerebellar cortex [32]. 

In humans, the work of Bullitt and collaborators clarified the role of physical exercise in angiogenesis [33]. The authors investigated cerebral circulation using Magnetic Resonance Angiography (MRA). They observed that active older adults (64 ± 5 years) who do at least 180 min of physical activity per week at a moderate intensity (55–70% of their maximum heart rate) have a larger number of small vessels (diameter less than 0.5 mm) compared to older adults who are sedentary and perform less than 90 min per week of physical exercise [33]. This proliferation of blood vessels could be responsible for the increased regional cerebral blood flow observed following regular physical exercise. For example, using Arterial Spin Labeling and Magnetic Resonance Imaging, researchers studying older adults found that, after four months of aerobic training consisting of walking and stationary cycling (frequency: 2 × 40 min per week; intensity: somewhat hard), there was an increase in blood flow in the hippocampus, an area associated with memory function [34]. Another study demonstrated that an increase in blood flow in the hippocampus correlated positively with delayed logical memory changes after a training program [35]. This finding supports the cardiorespiratory hypothesis such that physical exercise induces an increase in cerebral blood flow, which ensures the availability of metabolic resources (oxygen and glucose) for achieving the best memory performance. However, angiogenesis is not the only mechanism that leads to an increase in cerebral blood flow. Another mechanism associated with cardiorespiratory hypothesis that is likely to play a role is vascular plasticity. 

Vascular plasticity can be defined as the ability of the blood vessel to recover its compliance under the effect of a stimulus such as physical exercise. The process by which physical exercise induces better arterial compliance has been investigated by a recent study done in rodents [36]. The authors have shown that in rats, regular physical exercise leads to the bioavailability of nitric oxide (NO), which is an enzyme that plays a vasodilating role. The authors suggest that this bioavailability of NO will inhibit the activity of transglutaminase, which is the main agent of vessel stiffness. This would improve the vasodilator function of blood vessels. In humans, the mechanism of vascular plasticity has been investigated by Tanaka and collaborators with ultrasound imaging and applanation tonometry [37]. Using both devices, the authors have measured the diameter of the arteries and pressure waveform and amplitude. Based on these data, arterial compliance (see [38]) and β stiffness index (see [39]) have been calculated. The authors found, in their cross-sectional study, that older adults who have higher cardiorespiratory fitness have a significantly higher compliance of blood vessels than sedentary older adults. In addition, their study demonstrated that cardiorespiratory fitness correlated positively with compliance of blood vessels (*r* = 0.44). This positive correlation demonstrates that compliance and cardiorespiratory fitness move in the same direction for older adults. In their second, interventional study, the same authors found that after a 13-week training program, the compliance of blood vessels of older adults increased significantly. The characteristics of this program were: 73% of maximal heart rate (Intensity) for 42 min/day; 5 day/week for 13 weeks. It is clear from this work that improving the compliance of the blood vessels likely contributes to the increase in cerebral blood flow following the practice of physical exercise, partially supporting the cardiorespiratory hypothesis. However, this study did not examine the relationship between increased cerebral blood flow and cognitive performance. 

Similar to rodent findings, the increase in blood vessel compliance observed in humans can also be explained by the better availability of NO. The effect of NO on blood vessels in humans is a function of the acute or chronic nature of exercise [40]. Indeed, acute physical exercise induces transient vasodilation of the blood vessel, while chronic physical exercise is associated with remodeling of the blood vessel, which results in increased diameter of the lumen of the blood vessel [38]. In both cases, this leads to a better compliance of the blood vessels and, therefore, allows for an improvement in vascular health. For example, it has been demonstrated that six months of aerobic physical exercise is enough to improve vascular structure and function in African Americans [41]. A systematic review including 21 randomized controlled studies confirmed that aerobic exercise alone or combined with resistance exercises induce significant reductions (standard mean difference of −0.52 ms^−1^) in the stiffness of the arteries [42]. This has important implications for the maintenance of cognitive function, as stiffness in the arteries contributes to cognitive decline [43].

**Figure 1:**
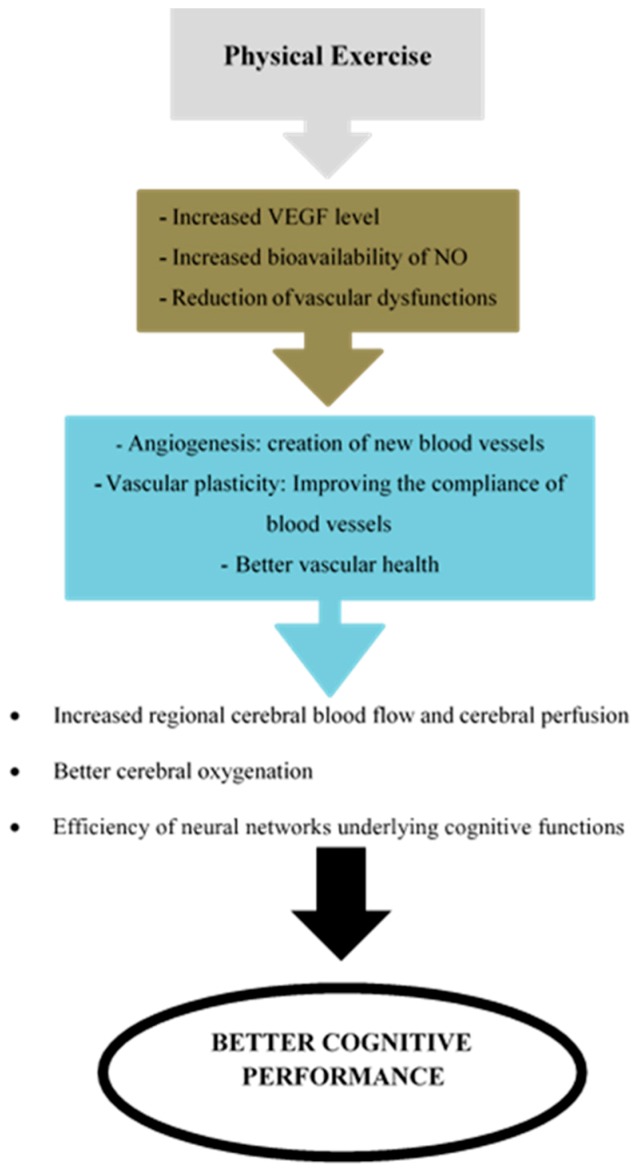
Main mechanisms of the cardiorespiratory hypothesis. VEGF = Vascular Endothelial Growth Factor; NO = Nitric Oxide.

Finally, the last mechanism associated with the cardiorespiratory hypothesis is the improvement of vascular health. In addition to the impacts of physical exercise on arterial stiffness, exercise can also reduce vascular dysfunctions that can occur with advancing age [44]. Among these dysfunctions, there are atheromatous plaques [45] that promote arterial hypertension and apoptosis of endothelial cells [46]. Aerobic exercise reduces blood pressure by reducing vascular resistance and thus reduces the risk of cardiovascular disease [47], an important contributor to cognitive decline [48]. Moreover, physical exercise can also reduce endothelial cell apoptosis through its positive action on endothelial cell telomerases [49]. All these mechanisms underlying the cardiorespiratory hypothesis are summarized in Figure 1. 

In addition to aerobic training, other studies have shown the effects of resistance training on the mechanisms underlying the cardiorespiratory hypothesis. For example, recent studies have demonstrated that resistance training increases vascular structure and function [50], vascular endothelial function, and peripheral blood circulation [51] and angiogenesis [52]. However, this effect of resistance training seems to depend on the intensity of training. A recent review demonstrated that in comparison to low intensity training, vigorous resistance training increases arterial stiffness [53]. Despite these effects of resistance training on vascular structure and angiogenesis, studies have supported the neurotrophic hypothesis (and not the cardiorespiratory hypothesis) to explain the prophylactic effects of resistance training on cognitive functions [54,55]. The neurotrophic hypothesis suggests that “*endogenous proteins* (brain-derived neurotrophic factor, IGF-1, fibroblast growth factor) *that support brain plasticity likely mediate the beneficial effects of exercise on the brain*” p. 236 [56].

The studies described above, which focused on the explanatory mechanisms of the cardiorespiratory hypothesis, were based on microscopic processes. However, given the complexity of cognitive and cardiorespiratory functions, it is now suggested that the exploration of these mechanisms should be carried out using other approaches, such as the non-linear model (see [57]) or the complex systems methodologies (see [58]). Indeed, biological systems are inherently at diverse hierarchical and heterogeneous multilevel, requiring linking together different types of top-down and bottom-up modeling with various macroscopic and microscopic parameters (see [57]). 

## 3. Cardiorespiratory Hypothesis and Cognitive Decline

Our review revealed that there is no research examining the effect of cardiorespiratory fitness on MCI, dementia, and Alzheimer’s disease in the African continent. However, some relevant cross-sectional and interventional studies with older adults living in industrialised countries exist. The cross-sectional studies examined correlations between cardiorespiratory fitness and other variables such as brain health [59,60,61,62], cognitive function [63], reduced brain atrophy [64], and diminution of amyloid beta (Aβ) related effects on cognition [65]. The main findings of these studies support the cardiorespiratory hypothesis demonstrating that higher levels of cardiorespiratory fitness are associated with preserved cerebral grey matter and white matter, and better cognitive abilities in adults with AD. Further, increasing cardiorespiratory fitness has been associated with reduced brain atrophy and diminution of Aβ in AD [65]. In addition, there is some evidence that patients in the early stages of the disease show low cardiorespiratory fitness compared to non-affected patients [66]. In contrast, higher cardiorespiratory fitness is associated with a low risk of death in AD patients [67]. All these results show that cardiorespiratory fitness may play an important role in the care of AD in any older population. 

Apart from these cross-sectional studies, other interventional studies have examined the prophylactic effects of a higher level of cardiorespiratory fitness following aerobic exercise in patients with MCI and AD. In the case of MCI, the results of these studies demonstrate that six-months of high-intensity aerobic exercise improves executive control processes (i.e., planning, inhibiting, and switching) in older women with MCI [68]. Similarly, a 12-week moderate intensity training program improved peak rate of cardiorespiratory fitness and this was associated with widespread increases in cortical thickness in MCI patients [69]. When examining individuals with AD, the results of a pilot exercise intervention study suggests that patients with AD are able to improve their cardiorespiratory fitness [70]. Following this pilot study, two randomized controlled trials demonstrated the positive effect of aerobic exercise on physical performance [71] and functional ability in patients with AD [72]. In addition, cardiorespiratory gains were associated with improved memory performance and reduced hippocampal atrophy [72]. The details of these interventional studies are presented in Table 1. These interventional studies in older adults partially validate the cardiorespiratory hypothesis. Indeed, they show that high cardiorespiratory fitness was associated with an improvement in executive performance [68,72]. In addition, the results show an increase in IGF-1 involved in angiogenesis after aerobic training [68]. However, interventional studies did not examine the links between cardiorespiratory fitness, executive performance, and neurophysiological correlates such as cerebral oxygenation. It seems essential that further research is done in this area in order to fully support the cardiorespiratory hypothesis. 

Other studies have investigated the effect of resistance training in patients with cognitive decline and showed mixed results. Some studies have demonstrated improvements in cognition after resistance training without explaining the underlying mechanisms [73,74]. Conversely, another study failed to demonstrate this effect of resistance training [75]. Although resistance training does improve some of the components of the cardiorespiratory hypothesis (angiogenesis (see [52]) and vascular health (see [50,51])), improvements in cognition after resistance training have typically been associated with the neurotrophic hypothesis [54,55]. Therefore, it would be interesting that future studies test the cardiorespiratory hypothesis after a resistance training program.

However, it is worth noting a limitation in relation to the research reviewed in this section. Indeed, whether in cross-sectional or interventional studies, cardiorespiratory fitness was measured with aerobic physiological markers like peak oxygen uptake (VO_2peak_) or maximal oxygen uptake (VO_2_max). These physiological markers of cardiorespiratory fitness, which measure metabolic, cardiorespiratory, and pulmonary responses during exercise, provide little information on the coordination between these three subsystems [76]. In order to overcome this problem, recent studies have developed an alternate way of measuring cardiorespiratory function: Cardiorespiratory coordination [76,77]. This approach applies a principal component analysis on several physiological parameters (expired fraction of O_2_, expired fraction of CO_2_, ventilation, systolic blood pressure, diastolic blood pressure, and heart rate) measured during an exertion test. The principal component analysis allows for the identification of the component that explains the greatest variation in the physiological parameters included in the analysis [76,77,78]. Thus, the studies reviewed above, which did not use this technique to explore cardiorespiratory function, are unable to provide information on co-variations of the cardiovascular and respiratory systems. As suggested by the authors of references [76,77,79], using the cardiorespiratory coordination approach should be more sensitive to training effects than the traditional aerobic physiological markers, and thus could be advantageously used in cross-sectional and longitudinal protocols. Despite this, its feasibility and validity for use in older populations must be demonstrated first. 

## 4. Future Research Directions

The results of our narrative review revealed that the studies that have investigated the effects of cardiorespiratory fitness on cognitive decline and pathological aging in Africa are non-existent. Similarly, data on African Americans with AD, dementia, or MCI regarding advantageous effects of cardiorespiratory fitness on cognition are also lacking [80]. However, one study conducted among African Americans without AD, dementia, or MCI demonstrated a relationship between poor cardiorespiratory fitness and poor cognitive performance [81]. The lack of studies in the African continent could be explained by the fact that age-related problems are not an immediate priority for decision makers or the African population. Indeed, the current priorities are focused on the problems of young people (unemployment, education). As the proportion of older adults within this population increases along with the number of cases of cognitive decline, this area will become an increasing priority.

In contrast, the ability of cardiorespiratory fitness to influence cognitive performance, physical ability, and brain structure in Caucasians with MCI or AD have been supported in interventional studies (see Table 1). Based on these studies, it is imperative that researchers initiate research projects aimed at replicating the findings reported in this narrative review and to identify, if any, potential specificities related to the relationship between the cardiorespiratory hypothesis and cognitive decline in the African population. As life expectancy increases in Africa and the continent faces higher non-communicable disease rates [16] and increasing incidence of cognitive decline (MCI, dementia, AD), it will be important for researchers to evaluate how the cardiorespiratory hypothesis applies to this population. For example, a longitudinal study could examine the effect of a physical training program on cardiorespiratory fitness parameters and the prevalence and incidence of age-related diseases. Based on the intervention studies reviewed, characteristics of the program could be: (a) Training time per week: Between 60 and 240 min; (b) total duration of the intervention program: For at least six months; (c) frequency: Between three and five sessions; intensity: Should vary from 40% to 85% of Heart Rate Reserve (HRR) if possible [68,69,70,71,72].

One of the interventional studies in our narrative review [72] demonstrates that a physical exercise program can induce an increase in cardiorespiratory fitness that correlates with memory performance in older adults with AD. This effect of physical exercise on memory could be explained by the mechanisms underlying the cardiorespiratory hypothesis, namely angiogenesis and the improvement of vascular plasticity. Another avenue for investigation could, therefore, be exploring the effects of physical exercise on angiogenesis and/or vascular plasticity in the elderly with AD, dementia or MCI in African nations. The objective would be to replicate the effect of physical exercise on angiogenesis and/or vascular plasticity in African older adults with AD, dementia, or MCI. Although data on the effects of cardiorespiratory fitness on aging-related diseases are lacking in Africans, results should be consistent with those observed in Caucasian populations after controlling for other factors (e.g., age, education, etc.).

It is well known that the mechanisms of angiogenesis and vascular plasticity allow the increase of cerebral blood flow and, consequently, the increase in cerebral oxygenation. Another possible direction could be the investigation of the cerebral oxygenation of AD, dementia, and MCI patients following regular physical activity. The cerebral oxygenation of patients could be investigated with cutting edge portable imaging tools such as functional near-infrared spectroscopy (fNIRS). fNIRS is a relatively low-cost optical imaging technique that allows for the investigation of hemodynamic changes associated with brain activity and has been successfully used to examine activations of the cerebral cortex in normal aging [82] and in patients with AD [83]. Moreover, this optical neuroimaging technique has been proved to be sensitive to brain activation differences as a function of cardiorespiratory fitness in older adults [84]. In addition, it has been shown that this tool can be used in the African population to monitor cerebral hemodynamic activity [85,86]. This technique could, therefore, be used to establish additional evidence of the cardiorespiratory hypothesis by measuring the hemodynamic activity of the cerebral cortex before and after a physical exercise program in African older adults with MCI, dementia, or AD. 

Finally, following the same rationale as the limitations outlined at the end of Section 2 on the complexity of the biological systems, because the human organism can be seen as an integrated network, a new field of exploration called the network physiology has been developed [87,88]. This field uses a network approach to better understand “*how diverse physiological systems and organs coordinate their functions over a broad range of space and time scales and horizontally integrate to generate distinct physiologic states at the organism level*” p. 1 [88]. A last potential direction of research could be to explore the nature of the links and whether there are mediating or moderating pathways that exist between the cardiac (heart rate, heart rate variability), respiratory (breathing rate, breathing variability), and central nervous (brain activity, brain variability) systems in patients with neurodegenerative diseases. Subsequently, the effects of physical exercise on the functional connectivity between the three systems could be examined (see [89], chap. III and IV).

## 5. Conclusions

This review highlights the absence of studies on the effects of cardiorespiratory fitness on cognitive performance in patients with MCI or AD in the African continent. However, there are some studies in industrialized countries on Caucasians and African Americans that highlight the possible beneficial effects of cardiorespiratory fitness on the physical and cognitive performances of older individuals with cognitive decline or pathological aging. Based on these results, we have proposed some potential avenues of research that should be developed for the African continent. Future studies examining these avenues of research could lead to a better strategy for the prevention of MCI, dementia, and AD in Africa prior to a major increase in the older population. To adopt these strategies, it seems essential that the area of geriatric medicine develops and fosters research into the cardiorespiratory hypothesis in aging and disease as an important area of inquiry, as aging is becoming widespread in all the countries of the continent.

## Figures and Tables

**Table 1:** Overview of interventional studies in patients with MCI or AD.

References	Characteristics of Training	Subjects	Measures	Main Results
[68]	Aerobic exercise: 4 sessions per week. The duration of the session varies between 45 and 60 min. The intensity of training increased from 75% to 85% HRR. Subjects of the control group carried out stretching and balance with an intensity that is less than 50% of the heart rate reserve. The duration of the training is six months.	33 subjects (mean age 70 years) diagnosed with amnestic MCI were enrolled in the study. However, 19 subjects of the aerobic group and 10 subjects of control group included in analysis.	CRF, executive function and memory tests and plasma IGF-1	CRF increased only in the aerobic exercise group. High-Intensity aerobic exercise improved the performance of multiple tests of executive function in women. However, for men, only the performance of the Trail-making test B has increased. Plasma IGF-1 increased after aerobic exercise intervention specifically in men.
[69]	Aerobic exercise: 120 min per week, spread over four sessions. The intensity of aerobic exercise increased from 50% to 60% of HRR. The duration of the training is three months.	14 subjects with MCI (78.85 ± 7.75 years) and 16 healthy subjects (75.87 ± 6.9 years) were enrolled in the study.	Changes in CRF, cortical thickness with MRI	CRF increased from pre to post intervention in both groups. The increase in CRF was associated with widespread increased cortical thickness.
[70]	Aerobic exercise: Three sessions per week. The duration of the session was progressively increased to reach 45 min. The intensity of training is moderate and was evaluated by perceived exertion rating. The duration of the training is six months.	Eight subjects with AD (81.4 ± 3.58 years) were enrolled in the study.	CRF, lower extremity function with short physical performance battery	Subjects with AD are capable of participating in aerobic exercise intervention and appear to improve their CRF.
[71]	Aerobic exercise: 180 min per week, spread over three sessions; intensity: from 70% to 80% of maximal heart rate. The control group received usual care during the intervention period. The duration of the training is four months.	After the exclusion criteria, 200 subjects with AD were enrolled. 107 in aerobic exercise group (69.8 ± 7.4 years) and 93 in control group (71.3 ± 7.3 years).	Test of physical performance, test of dual-task performance (walk and naming the months backwards starting with January (dual-task month), test of exercise self-efficacy and tests of cognition and neuropsychiatric symptoms	Aerobic exercise improved potentially CRF single-task physical performance, dual-task performance, and exercise self-efficacy in patients with mild AD.
[72]	Aerobic exercise: 60–150 min per week, spread over 3–5 sessions; intensity: From 40–55% to 60–75% of HRR. Stretching and toning control group: Non-aerobic exercises with an intensity below 100 beats per minutes. The duration of the training is six months.	After the exclusion criteria, 76 subjects with probable AD were enrolled. 39 in aerobic exercise group (74.4 ± 6.7 years) and 37 in control group (71.4 ± 8.4 years).	Cognitive test battery, depression and functional ability, CRF, hippocampal and total gray matter volume with MRI (magnetic resonance imagery)	Aerobic exercise was associated with a modest gain in functional ability. Exercise-related gains in CRF were associated with improved memory performance and reduced hippocampal atrophy.

AD: Alzheimer Disease; CRF: Cardiorespiratory Fitness; MCI: Mild Cognitive Impairment; IGF-1: Insulin Growth Factor-1; HRR: heart rate reserve.
